# Hereditary Nonpolyposis Colorectal Cancer and Cancer Syndromes: Recent Basic and Clinical Discoveries

**DOI:** 10.1155/2018/3979135

**Published:** 2018-04-23

**Authors:** Erbao Chen, Xiaojing Xu, Tianshu Liu

**Affiliations:** Department of Medical Oncology, Zhongshan Hospital, Fudan University, Shanghai, China

## Abstract

Approximately one-third of individuals diagnosed with colorectal cancer have a family history of cancer, suggesting that CRCs may result from a heritable component. Despite the availability of current gene-identification techniques, only 5% of all CRCs emerge from well-identifiable inherited causes for predisposition, including polyposis and nonpolyposis syndromes. Hereditary nonpolyposis colorectal cancer represents a large proportion of cases, and robustly affected patients are at increased risk for early onset, synchronous, and metachronous colorectal malignancies and extracolonic malignancies. HNPCC encompasses several cancer syndromes, such as Lynch syndrome, Lynch-like syndrome, and familial colorectal cancer type X, which have remarkable clinical presentations and overlapping genetic profiles that make clinical diagnosis a challenging task. Therefore, distinguishing between the HNPCC disorders is crucial for physicians as an approach to tailor different recommendations for patients and their at-risk family members according to the risks for colonic and extracolonic cancer associated with each syndrome. Identification of these potential patients through epidemiological characteristics and new genetic testing can estimate the individual risk, which informs appropriate cancer screening, surveillance, and/or treatment strategies. In the past three years, many appealing and important advances have been made in our understanding of the relationship between HNPCC and CRC-associated syndromes. The knowledge from the genetic profile of cancer syndromes and unique genotype-phenotype profiles in the different syndromes has changed our cognition. Therefore, this review presents and discusses HNPCC and several common nonpolyposis syndromes with respect to molecular phenotype, histopathologic features, and clinical presentation.

## 1. Introduction

Colorectal cancers (CRCs) are one of the most common malignancies and represent the third most common cancer in men and the second in women worldwide. It is estimated that one-third of individuals diagnosed with colorectal cancer have a family history of cancer [1]. However, approximately 5% to 10% of all CRCs, called hereditary CRCs, are related to mutations and defects in certain genes [2]. In addition, approximately 20–30% of familial CRC patients have at least one relative affected by neoplasms that act as nonsyndromic familial CRCs and are likely driven by shared genes and/or environmental factors [3, 4]. In individuals, high-risk hereditary predisposition syndromes have been associated with a 70–100% lifetime risk for earlier development of CRCs or metachronous cancers, and many syndromes carry an increased risk for extra-intestinal manifestations [5].

The hereditary CRCs syndromes are broadly divided into nonpolyposis and polyposis syndromes. In 1966, Lynch first defined familial CRC type I for families with CRC only and type II for families with both CRC and gynecological cancer [6]. Later, the term hereditary nonpolyposis colorectal cancer (HNPCC) was recommended to emphasize the absence of a polyposis phenotype. Currently, HNPCC defines a patient who meets Amsterdam criteria I or II [7–9], and HNPCC patents are prone to develop synchronous and metachronous cancers at relative young ages. Generally, the “nonpolyposis” label of HNPCC can be misleading and confusing to physicians, because adenomas typically present a villous growth characteristic and have different degrees of cell dysplasia [10]. Therefore, identification of these individuals is critical for early intervention and treatment of associated malignancies to reduce HNPCC-associated morbidity and mortality. Most of the cancer syndromes involve inherited mutations in genes that modulate growth processes in colonic stem cells and/or protect the fidelity of the genome passed into progeny cells. Given the substantial risk of synchronous and metachronous cancer, these mutation carriers are recommended to abide by standard surveillance and comprehensive management protocols when compared with the general population with an average CRC risk profile. The role of genetic counseling becomes crucial in treating these patients.

As such, understanding and distinguishing the various syndromes are useful and clinically meaningful as the approach to diagnosing and surveilling patients and their at-risk family members. Previously, testing of patient tumors for microsatellite instability (MSI) and mutation of DNA MMR genes is an effective strategy to discriminate patients at risk for Lynch syndrome. Cancer-free individuals whose family history indicates suspicion for a hereditary cancer syndrome should undertake clinical genetic evaluation and receive genetic counseling. Even if a mutation is not detected, people may benefit from the genetic evaluation in other interventions to reduce future cancer risk.

**Figure 1:**
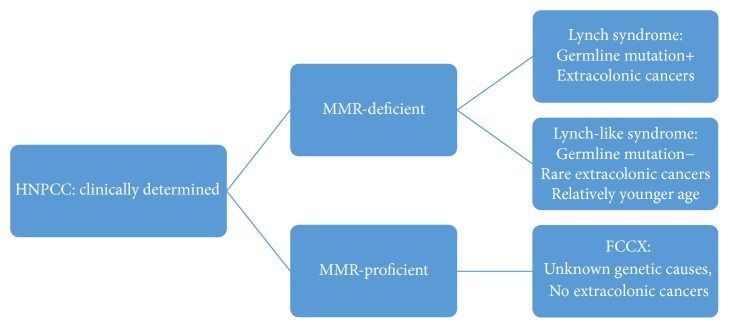
The category of hereditary nonpolyposis colorectal cancer.

This paper provides a comprehensive literature review on the most common HNPCC and HNPCC-associated cancer syndromes (including Lynch syndrome, Lynch-like syndrome, and familial colorectal cancer type X) that present distinguishing molecular phenotypes, histopathologic features, and clinical presentations among different subtypes (Figure 1).

## 2. Diagnosis of Hereditary Nonpolyposis Colorectal Cancer

The Amsterdam I clinical criteria for HNPCC, which focus on the number and ages of family members with colorectal cancer, were published in 1990 to standardize the inclusion criteria for clinical research studies [9]. For kindred families fulfilling the Amsterdam criteria, the chance of identifying a germline mutation is 40% to 50% [11]. Similarly, 40% of patients with an identified genetic mutation fail to meet Amsterdam criteria [12]. Therefore, Amsterdam I criteria were believed to be insufficiently sensitive and to be missing clear familial clustering of extracolonic malignancies, which led to establishment of the Amsterdam II criteria in 1999, which improved the diagnostic sensitivity and included associated cancers (e.g., endometrial, small bowel). Later, Bethesda criteria were created and revised in 2004 to identify CRC patients who should undergo pathologic examination (MSI and/or immunohistochemistry (IHC) assessment for the MMR protein deficiency in the tumor) for HNPCC/Lynch syndrome [13] (Box 1).

**Figure 2:**
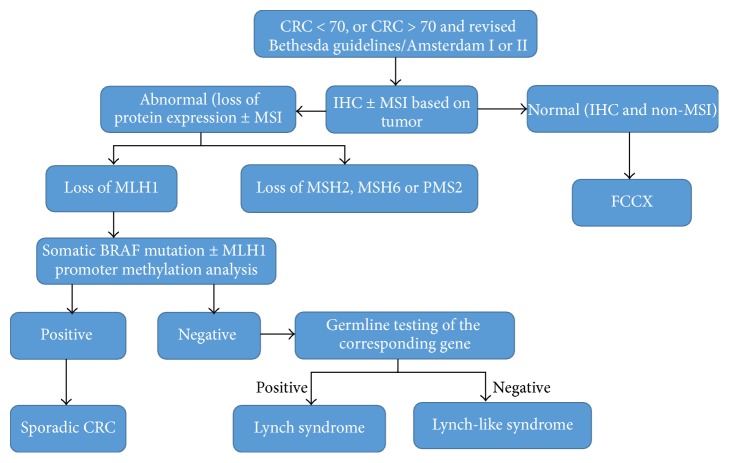
The diagnostic algorithm for HNPCC-associated cancer syndrome.

Regardless, the Amsterdam criteria fail to identify approximately 50% of cases, and Bethesda guidelines fail to identify at least 30% of cases [14], which has led to increased support for the universal application of the polymerase chain reaction (for detection of MSI-high tumors) and/or IHC testing to all CRC specimens [15]. Currently, many guidelines suggest two possible approaches to screen out Lynch syndrome: a universal one, that is, to test every patient with CRC, and a selective one (Jerusalem guidelines), which broadens the indications for MSI or IHC testing to every individual with CRC diagnosed prior to age 70 plus patients diagnosed at older ages who meet the Bethesda criteria [16], with the latter approach missing more than a quarter of patients with Lynch syndrome (Figure 2). This justification also supports the universal testing for endometrial cancer [17]. Universal testing followed by germline testing offers the highest sensitivity (and somewhat lower specificity) than alternative screening strategies, although the increase in the diagnostic yield is modest compared with criteria-based screening techniques [18]. Cost-effectiveness analyses demonstrate varying results [19, 20]. In this review, HNPCC generally refers to any family that meets the Amsterdam I/II or Revised Bethesda guidelines.

## 3. Lynch Syndrome

### 3.1. Overview

One of the first hereditary nonpolyposis cancer-associated syndromes to be identified, Lynch syndrome, is also the most relevant to HNPCC. It is estimated that LS accounts for approximately 3% of all CRC cases, and its prevalence in the general population is one in 440 [21]. Lynch syndrome also increases the risk for extracolonic cancers such as that of the endometrium (50–60%), ovaries (9–14%), stomach (13–19%), small intestine, urinary tract, and central nervous system [22, 23] (Table 1). Lynch syndrome is now well established as an inherited, autosomal dominant predisposition to CRC, and certain extracolonic cancers are derived from defective DNA mismatch repair (MMR) genes, which are a system for maintaining genome integrity. Accumulating genetic mutations of MMR genes lead to tracts of repetitive DNA sequences called microsatellites, which typically manifest microsatellite instability (MSI) [24]. MSI facilitates tumor cell proliferation, invasion, and metastasis through activating oncogenes or suppressing tumor suppressors [25] and allows detection of microsatellite instability (MSI) during tumor progression [26].

### 3.2. The Molecular Phenotype of Lynch Syndrome

Lynch syndrome is an autosomal dominant condition caused by a malfunctioning MMR system resulting from the pathological mutation in at least one of the MMR genes. Germline testing for germline mutations in the MMR genes—mutS homologue 2 (MSH2), mutL homologue (MLH1), mutS homologue 6 (MSH6), and postmeiotic segregation increased 2 (PMS2)—can discriminate Lynch syndrome from Lynch kindreds. During cellular proliferation and differentiation, DNA randomly produces errors that may include single base mismatch, insertions, misincorporation, and deletion of bases. The function of the MMR gene system is to recognize and maintain the fidelity of DNA by correcting the structure of DNA replication errors [27]. Approximately 70–90% of Lynch syndrome tumors have germline MLH1 or MSH2 mutations. MLH1 and MSH2 mutations confer an elevated lifetime cancer risk when compared to MSH6 and PMS2 [28, 29]. MSH6 and PMS2 are detected in the remaining approximately 10–20% of LS cases, and up to 3% of LS is caused by deleterious mutations in epithelial cell adhesion molecule (EPACM). The deletion of the 3′ end of EPCAM immediately upstream of MSH2 on chromosome 2, which causes allele-specific methylation of the MSH2 promoter, is a relatively rare cause for epigenetic silencing of MSH2 [30]. Somatic BRAF mutations can be useful to rule out Lynch syndrome, because they frequently occur in sporadic MSI CRCs, caused by MLH1 promoter methylation. BRAF and promoter methylation are usually conducted in patients with a lack of MLH1 protein to eliminate the possibility of Lynch syndrome.

Recent studies have focused on exploring the novel somatic mutations in the Lynch syndrome. For instance, RNF43, one of the E3 ubiquitin ligases, acts as a negative-regulatory factor of the WNT pathway via reducing membrane surface expression [31]. RNF43 had been regarded as a pivotal mutational target of microsatellite instability (MSI) in 70–80% of sporadic colorectal carcinogenesis [32]. Similarly, RNF43 mutations occurred in approximately 40% of LS-CRCs, which was significantly lower than the percentage occurring in sporadic colorectal cancers [33]. Additional somatic mutations of POLE and MSH3, identified in LS-CRCs, are regarded to further accumulate somatic mutations during tumor progression [34]. Constitutional MMR deficiency (CMMRD), a very rare subtype of MMR deficiency, is a distinct childhood cancer predisposition syndrome characterized/caused by the presence of MMR homozygous mutations or inherited biallelic MMR protein inactivation [35]. The CMMRD harbors more somatic mutations compared with Lynch syndrome individuals, especially in the DNA-binding domain of the TP53 gene. Some novel mutations such as POLE and POLD1 have also been identified in the CMMRD [36]. Female POLD1 mutation carriers have a high risk of endometrial cancer and a moderate risk of breast cancer [37] (Table 2).

### 3.3. The Histopathologic Features and Clinical Presentation of Lynch Syndrome

Since Lynch syndrome has been extensively characterized, however, the incidence of Lynch syndrome varies by the endemicity of all diagnosed CRC patients in different populations. Similar to the case of European individuals with Lynch syndrome [38], recent clinic-based observations have reported deleterious mutations that disrupt the function of the MMR gene product (MLH1 (61%), MSH2 (21%), PMS2 (12%), and MSH6 (6%)) in an African American population with Lynch syndrome [39]. The percentage of MMR gene mutations in the tumors was approximately 13% in Latino individuals, which is similar to estimates in non-Hispanic white individuals. Furthermore, approximately 61.9% of deficient MMR tumors were indeed attributable to germline MMR gene mutations by in-depth molecular analysis, and Latino patients with Lynch syndrome develop cancer at a younger age and have a higher percentage of rectal cancers and advanced disease, which is consistent with observations in other studies [40]. The incidence of Lynch syndrome among diagnosed CRC patients in Japan is 0.7%, which is slightly lower than that reported previously but within the same range (0.7–3.7%) as that in recent investigations [41]. The CRC incidence in Finnish MLH1 mutation carriers was lower than that in non-Finnish carriers, but not significantly [42].

Very few studies have compared the epidemiological characteristics and clinicopathological differences between Lynch syndrome patients with other disease and those with Lynch syndrome only. The MSH6 and PMS2 mutations indicate a decreased risk of cancer incidence when compared with MLH1 and MSH2 mutations [28]; however, when comparing individuals with breast cancer only to those with CRC only, MSH6 and PMS2 mutations were more frequent than MLH1 and MSH2 mutations [43]. Inflammatory bowel disease (IBD) is associated with a 1.7-fold greater CRC risk (95% confidence interval, 1.2–2.2) due to inflammatory factors [44]. In patients with both Lynch syndrome and IBD, there is an increased CRC risk at a younger onset compared to those with Lynch syndrome only, especially in patients with ulcerative colitis [45, 46].

Screening by colonoscopy enables the early detection and removal of preinvasive neoplasia or adenomas before the presence of symptoms and is the main strategy of secondary prevention in Lynch syndrome patients [47, 48]. Although the removal of adenomas is regarded as an effective way to prevent CRC and death, CRC still occurs frequently. However, the overall survival is good for patients with CRC and for patients with first endometrial and ovarian cancer [49]. The outcomes for Lynch syndrome patients who have survived a first cancer attack are of great interest. In patients who have their first cancer before the age of 40, the cumulative incidences for any subsequent cancer are high, specifically, 73% for MLH1 mutations, 76% for MSH2 mutations, and 52% for MSH6 mutations [50]. Of note, the relative incidence of subsequent cancer compared with the incidence of first cancer is slightly but insignificantly higher than the cancer incidence in patients with Lynch syndrome without previous cancer. MSH2 mutation patients tend to have prostate and urinary tract cancers. Upper gastrointestinal tract cancers frequently occur in MLH1 mutation carriers, whereas ovarian cancer occurs mainly in younger women, which is contrary to ovarian cancers in BRCA1/2 mutation carriers and in the general population [51].

For treatment, MSI in a tumor usually causes frameshift mutations in the DNA, resulting in immunogenic neoantigens that induce an immune reaction against the tumor. The approval of pembrolizumab use for solid tumors with high-level microsatellite instability or mismatch repair deficiency by the US Food and Drug Administration highlights the promise of precision immuno-oncology. Immunotherapy strategies are based on immunopathology. Hence, patients would benefit from tumor immunopathology, especially for some immune checkpoints, such as PD1 (programmed cell death 1) and PDL1 (programmed cell death 1 ligand 1).

## 4. Lynch-Like Syndrome

### 4.1. Overview

As many as 60–70% patients who fulfill the AC criteria in the clinical and MMR deficiency in the tumor but for whom germline testing lacks a detectable germline mutation are defined as having “Lynch-like syndrome” [52]. Due to an early-onset age and abnormal MMR protein similar to those of Lynch syndrome patients, Lynch-like syndrome patients are nearly impossible to differentiate from Lynch syndrome patients. They all manifest MSI within their cancers, and immunohistochemistry detects abnormal DNA MMR protein—not only for MLH1 but also for the main DNA MMR proteins, including MSH2, MSH6, and PMS2, as with true Lynch syndrome cancers [53]. Additionally, Lynch-like syndrome patients have a slightly higher but insignificant mean age of onset than that of Lynch syndrome patients (53.7 versus 48.5 years of onset). The only differentiating clinicopathological features between these two cancer syndromes are as follows. (1) The majority of those with Lynch-like syndrome tend to have CRC in the right colon (93%) when compared to those with Lynch syndrome (45%) [54]. (2) Epidemiologic studies to date have described lower standardized incidence ratios for colorectal cancer (2.12 versus 6.04) and noncolorectal cancer Lynch syndrome-associated cancers (1.69 versus 2.81) in Lynch-like syndrome compared with Lynch syndrome. Lynch-like syndrome is estimated to account for as many as 70% of clinical Lynch syndrome patients suspected to have a high MSI condition and the absence of MMR proteins. Consequently, clinicians are limited in their knowledge of the genetic diagnosis of these patients and are unconfident regarding which screening should be recommended [55].

### 4.2. The Molecular Phenotype of Lynch-Like Syndrome

The mechanism between Lynch syndrome and Lynch-like syndrome for causing the generation of MSI within Lynch-like syndrome patients is appealing and elusive. Three hypotheses have been proposed to explain why Lynch-like syndrome patients show MSI in the tumors but no DNA MMR germline mutation: (1) there exist unknown gene mutations other than the DNA MMR genes in the germline that can drive MSI. Lynch syndrome and Lynch-like syndrome CRC frequently harbor not only activating ERBB2 mutations but also specific mutational patterns in PIK3CA and KRAS. Furthermore, ERBB2-mutated MSI CRC is susceptible to irreversible pan-HER blockade [56]. Recently, MCM9 was identified as the DNA helicase in the MMR complex, and loss of helicase activity results in MSI. MCM9 recruitment and loading into chromatin are MSH2-dependent and strengthens the recruitment of MLH1 to chromatin binding sites, and cells lacking the MMR protein lose the maintenance of genome stability [57] Liu et al. reported that MCM9 unknown significance variants were only observed in a small proportion of Lynch-like syndrome patients and that MCM9 mutations are unable to explain the MSI in most Lynch-like syndrome cases [58]. (2) Limited methods failed to detect germline mutations in the DNA MMR genes [59]. A new method of allelic dropout in long PCR was performed to seek potential regions of rearrangement in the MSH2 gene. Six of 10 (60%) patients with previously unexplained MSH2-deficient Lynch syndrome were detected as harboring an inversion alteration from exon 1 to 7 in the MSH2 gene [60]. Traditional germline testing screens exons and splice sites for deleterious mutations but does not review introns or RNA transcripts for harmful alterations. cDNA screening to identify patients suspected to have cryptic MMR gene rearrangements is recommended [61]. (3) An unrevealed biological process within the tumors, not involving a germline mutation, for instance, a double somatic mutation in the MMR gene, mostly occurs in sporadic CRCs [62]. For instance, approximately 60% of Lynch-like CRCs manifest biallelic somatic inactivation of MMR genes within the tumor. A somatic mutation in one allele of an MMR gene along with loss of heterozygosity of the other allele is the most commonly described pattern. These somatic MMR gene mutations are likely sporadic events, suggesting that such tumors are most likely cancers with sporadic DNA MMR deficiency. Somatic mutation of POLE was identified as a rare possibly underlying cause for MMR deficiency in Lynch-like syndrome [63].

Other possible causes of Lynch-like syndrome-associated cancers could include false-positive results showing MSI. Each of these possibilities may be part of the reason for Lynch-like syndrome because they are not mechanisms that conflict with each other, and given the standardized incidence ratios of Lynch-like syndrome between the ratios for Lynch syndrome and sporadic colorectal cancer, Lynch-like syndrome may be a heterogeneous condition between these two extremes. In exploring the three hypotheses, the first regarding an unknown germline gene driving MSI is the most remote. The DNA MMR apparatus is well studied, and most associated components are known, with no other reports of germline mutations outside of these genes, other than EPCAM as described. Mensenkamp et al. made significant headway in identifying that somatic mutations in MLH1 and MSH2 are a frequent cause for inactivating DNA MMR function and subsequent MSI generation within Lynch-like syndrome cancers [64].

### 4.3. The Histopathologic Features and Clinical Presentation of Lynch-Like Syndrome

MUTYH encodes a base excision repair DNA glycosylase. Mutations in this gene are found in MUTYH-associated polyposis (MAP) syndrome, an autosomal inherited condition commonly featured by the presence of a few to hundreds of colonic adenomatous polyps and an increased CRC risk at a young age [65]. Biallelic MUTYH mutations also account for a proportion of LLS cases [66]. Patients with Lynch-like syndrome and Lynch syndrome caused by EPCAM deletion share common clinical features that differ from patients with Lynch syndrome caused by MMR, including a preference for the right colon, a lower degree of fulfillment of the revised Bethesda guidelines, and an older mean age at CRC diagnosis [63]. Patients with Lynch-like syndrome more frequently have colorectal carcinoma on the right side and are less likely to have synchronous or metachronous carcinoma. However, there are no significant differences in clinicopathological variables between patients with Lynch-like syndrome and Lynch syndrome with endometrial carcinoma [54].

## 5. Familial Colorectal Cancer Type X (FCCX)

### 5.1. Overview

HNPCC is a clinically heterogeneous disease of which approximately 4% of cases are associated with Lynch syndrome, which may not conform to AC, <1% of cases are correlated to a Lynch-like syndrome, and 2–4% of cases are diagnosed as FCCX [67]. Familial colorectal cancer type X is a collective designation by Lindor [68] and refers to families who fulfill AC I but exhibit no evidence of a deficient DNA MMR gene (no deleterious germline mutations in the MMR genes, no microsatellite instability, or no absence of immune-histochemical staining of MMR protein), wherein “X” is used to describe the unknown nature of the etiology. As is the case for other familial cancer syndromes, the identification of the genes associated with FCCX will facilitate the molecular diagnosis of the disease and the development of appropriate surveillance guidelines and clinical management protocols for these patients.

### 5.2. The Molecular Phenotype of FCCX

The genomic profiles of FCCX cancers show similarities to sporadic MMR-proficient CRC since the clinical performance and histopathological features resemble sporadic CRC. Chromosomal instability (CIN) is a type of genomic instability; as a result, the chromosomal structures are unstable and hence facilitate carcinogenesis. Recently, high-resolution genomic profiling elaborated a CIN-like profile that discriminated 65% of colorectal cancers associated with FCCX from LS based on the gain of chromosomal region 20q and loss of 18 [69]. The genomic profiles of FCCX cancers are very similar to those of stable early tumors but highly different from that of LS. Genome-wide linkage analysis suggests that linkage at four chromosomal regions, 2p24.3, 4q13.1, 4q31.21, and 12q21.2–q21.31, are responsible for FCCX cancers, and RASSF9 and NTS are considered good candidates because of their possible involvement in colorectal epithelium carcinogenesis [70]. Except for the linkage to certain chromosomal regions, genomic insertion/deletion (INDEL) and copy number variation (CNV) also locate parts or the entire sequences of genes and hence may regulate gene expression or function [71]. Some mutations in several cancer-related genes such as BMPR1a are also detected in FCCX, but an independent cohort of 22 probands from FCCX families have revealed that BMPR1a mutations are not a major contributor of FCCX incidence [72].

Moreover, previous studies suggest that mutations of the GALNT12 gene might elucidate the unknown etiology of familial CRC. However, investigators found that GALNT12 is not a major gene modulator in the predisposition to FCCX, when delineating the relevance of GALNT12 mutations in the etiology of FCCX [73]. BRCA2 is a putative tumor suppressor gene and plays a pivotal part in repairing DNA. Pathogenic mutations in germline DNA are predominantly responsible for hereditary breast and ovarian cancer. Four BRCA2 variants containing c.502C>A p. (Pro168Thr), c.927A>G p. (=), c.5744C>T p. (Thr1915Met), and c.7759C>T p. (Leu2587Phe) show cosegregation with FCCX and may exert a function as susceptibility alleles in FCCX families [74]. A truncating dominant negative mutation in SETD6 also provides a possible explanation for the cancer predisposition of one FCCX family [75]. Germline variants in the semaphorin 4A gene increase the predisposition to colorectal cancers in families with FCCX [76]. A single truncating germline mutation of RPS20, a ribosomal protein gene, was investigated in four generations of an FCCX family. This variant was associated with a fault in preribosomal RNA maturation and was considered a new colon cancer predisposition gene [77].

RASL10B encodes a small GTPase with antitumor properties, and epigenetic silencing of this gene has been attributed to hepatocellular carcinoma cells and breast cancer [78, 79]. RASL10B has been reported to be differentially hypomethylated in FXXC cancers compared to Lynch syndrome cancers [80]. RASL10B is one member of Ras superfamily with antitumor potential. Of note, segregation of deranged methylation of RASL10B was revealed in relation to the progression of sessile serrated adenomas/polyps5, which are regarded as a putative prelude of colon cancer. A number of studies have mainly focused attention on different genes between MMR-deficient and MMR-proficient nonpolyposis tumors. These two groups show various gene expression profiles of blood telomere length. Many studies support that long telomeres are associated with increased cancer risk: long telomeres may decrease cell apoptosis, accumulating deranged genomic aberrations. FCCX cancer patients had longer telomeres than healthy individuals of the same type X families and LS cancer families [81]. MMR-proficient and MMR-deficient tumors show diverse gene expression profiles in approximately 2000 significantly different genes. Functional enrichment pathways were involved in G-protein-coupled receptor signaling, proliferation and migration, cell cycle transition, DNA replication, and mitosis. Several candidate target genes such as NDUFA9, AXIN2, MYC, and H2AFZ have been specifically linked to FCCX tumors [82].

### 5.3. The Histopathologic Features and Clinical Presentation of FCCX

FXXC presents a special clinically different phenotype compared to that of Lynch syndrome families as follows [83]: (1) lower incidence of CRC; (2) developing CRC at a later age; (3) greater frequency in the distal colon; (4) poor differentiation and more mucinous characteristics; (5) distinctive morphological features, including tumor-infiltrating lymphocytes; and (6) fewer multiple tumors.

Obviously, FCCX is clearly easily differentiated from Lynch syndrome [84]. The identification of differences that distinguish patients with FCCX from those with sporadic CRC could reinforce the characteristics of the syndrome, but only the identification of the gene expression associated with FCCX will assist in the early diagnosis of the disease. Age at the diagnosis of cancer is significantly lower in FCCX than in sporadic CRC. Patients with FCCX have an approximately larger number of synchronous tumors, but this number does not reach the level of statistical significance. Recurrence is noticeably higher in FCCX than in sporadic CRC [85]. FCCX has a lower proportion of peritumoral lymphocytes and Crohn-like reactions. It is noteworthy that venous invasion is most commonly seen in FCCX [86].

## 6. Conclusions and Future Perspectives

To our limited knowledge, hereditary CRC syndromes are a few causes of CRC in the general population. Different cancer syndromes encompass a spectrum of similar clinical presentations and genetic profiles. These overlapping concepts and terms make early diagnosis in hereditary CRC cases clinically challenging. Therefore, an awareness and understanding of these unique syndromes may help early diagnosis and preventative interventions in individual patients and their family members. On the other hand, patients with cancer syndromes and their high-risk family members also expect early detection and intense surveillance to prevent and manage several life-threatening malignancies. Moreover, screening, genetic testing, and counseling of at-risk kindred can translate into a significant benefit across multiple generations, demonstrating the tremendous importance of understanding the genetic profile and clinicopathological features of each syndrome. We have herein provided a comprehensive overview of several common hereditary colorectal nonpolyposis and cancer syndromes for clinicians to successfully handle while engaged in a busy clinical practice. Physicians should therefore stay abreast of these discoveries.

There still remain some problems. Interactions with environmental factors make the identification and validation of genetic deleterious mutations or genes complicated. First, the human genome harbors a mass of rare variants, most of which may not be clearly or directly associated with disease phenotypes, and second, some of these alterations may interplay with other genetic and/or environmental factors, which may in turn influence their expression.

In the era of gene sequencing and other new molecular technologies, a number of new genes/mutations are being identified for the prevention and management of many cancers. These new methods will contribute to the investigation of unique genotype-phenotype profiles and may provide the treatment strategies based on individual risk in precision medicine.

## Figures and Tables

**Box 1:**
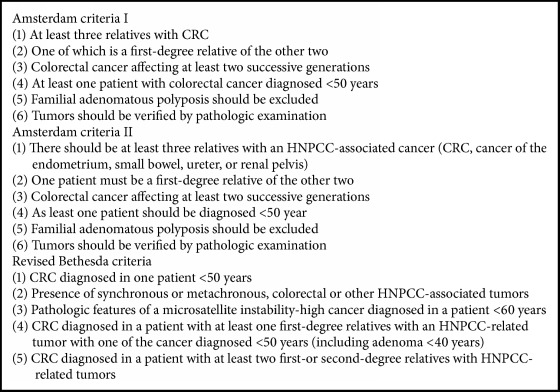
HNPCC clinical diagnostic criteria.

**Table 1:** Genes associated and cancer risks for HNPCC.

Syndrome	Gene	Percentage/syndrome	Cancer risks	95% CI
Lynch syndrome	MLH1	60%	Colorectum	52 (31–90)
			Endometrium	21 (9–82)
			Stomach	11–19
			Ovary	38 (3–81)
			Hepatobiliary	2–7
			Upper urinary tract	4-5
			Pancreas	3-4
			Small bowel	1–4
			Glioblastoma	1–3
Lynch syndrome	MSH2	20%	Colorectum	49 (29–85)
Lynch syndrome	MSH6	6%	Colorectum	18 (13–30)
			Endometrium	17 (8–47)
			Stomach	≤3
			Ovary	1 (0-1)
			Urinary tract	<1
Lynch syndrome	PMS2	12%	Colorectum	15–20
			Endometrium	15
Lynch syndrome	EPCAM	<3%	Endometrium	57 (22–82)
			Stomach	11–19
			Ovary	20 (1–66)
			Hepatobiliary	2–7
			Upper urinary tract	4-5
			Pancreas	3-4
			Glioblastoma	1–3

**Table 2:** Germline and somatic genetic and epigenetic characteristics of HNPCC-associated cancer syndrome.

	Lynch syndrome	Lynch-like syndrome	FCCX
Tumor MMR	MSI	MSI	MSS
Germline mutation	MSH2, MLH1, MSH6, PMS2, EPCAM	ERBB2, MCM9	RPS20, SEMA4A,
Somatic mutation	Second allele of MMR	Both alleles of a MMR	RASSF9, NTS, SETD6, NDUFA9, AXIN2, MYC, H2AFZ
Epigenetic phenotype	MSH2 promoter methylation	None	RASL10B

MSS: microsatellite stable; MSI: microsatellite instability; FCCX: familial colorectal cancer X; MMR: mismatch repair.
